# COGNITION ON THE GO: UNIQUE INSIGHTS INTO BRAIN HEALTH FROM HIGH-FREQUENCY COGNITIVE APPROACHES

**DOI:** 10.1093/geroni/igad104.2041

**Published:** 2023-12-21

**Authors:** Zita Oravecz, Laura Germine

## Abstract

High-frequency ambulatory cognitive assessments offer the opportunity to examine cognitive change at multiple timescales. When deployed multiple times per day via pervasive mobile technology (e.g., smartphones), ambulatory cognitive assessments allow us to conduct semi-continuous monitoring of cognitive changes while individuals go about their daily lives. Variation in performance that occurs over these short timescales may be associated with a wide range of contextual factors (e.g., our thoughts, emotions, and behaviors) including the way we respond to challenges presented by our everyday environments. Short-term change and variation may also reveal information about overall brain health and risk for cognitive decline over longer timescales. This symposium focuses on how high-frequency, ambulatory cognitive assessments can generate new insights into cognitive aging, psychological processes, and risk for neurocognitive decline by revealing detailed patterns of cognitive change and variation over short timescales (hours to weeks). The talk by Sliwinski et al. gives an overview of high-frequency cognitive assessment approaches. The talk by Harrington et al. will show how dimensions of experienced affect may influence short-term changes in multiple domains of cognition. The talk by Hakun et al. will show how short-term cognitive variation may predict how we respond to everyday stressful experiences. The talk by Oravecz et al. will show how to extract substantively meaningful predictors of long-term cognitive change from short-term processes such as retest learning.

